# Plant based Nano defenders successfully fight microbial contaminants without damaging the morphology and genetics of rose and night queen

**DOI:** 10.1371/journal.pone.0331792

**Published:** 2025-09-23

**Authors:** Ghulam Zahara Jahangir, Muhammad Zafar Saleem, Mubeen Akhtar, Rida Farooq, Ayesha Naz, Saima Younas, Maria Rafique, Rehan Sadiq Shaikh, Qurban Ali, Shiming Han, Daoud Ali

**Affiliations:** 1 Centre for Applied Molecular Biology, University of the Punjab, Quaid-e-Azam Campus, Lahore, Pakistan; 2 Department of Biotechnology, Superior University, Lahore, Pakistan; 3 Department of Biotechnology, Lahore College for Women University, Lahore, Pakistan; 4 Department of Plant Breeding and Genetics, Faculty of Agricultural Sciences, University of the Punjab, Lahore, Pakistan; 5 School of Biological Sciences and Technology, Liupanshui Normal University, Liupanshui, P.R. China; 6 Department of Zoology, College of Science, King Saud University, Riyadh, Saudi Arabia; University of Kashan, IRAN, ISLAMIC REPUBLIC OF

## Abstract

In this study, the callus of *Carica papaya* was treated with silver nitrate solution to form silver nanoparticles which were later used as anti-contaminant substances in micropropagation media on *C. nocturnum* (cestrum, night queen) and *Rosa indica* (rose) in tissue culture medium. For this purpose, the rose and cestrum explants were cultured using murashige and skoog (MS) media containing 2 mg/l benzylaminopurine (BAP). After micropropagation of the shoots, different concentrations of biogenically synthesized silver nanoparticles (AgNPs) i.e., 2, 3, and 5 ppm were added into the MS media, for dose optimization. The morphology of tissue-cultured plants in control and experimental has been studied and compared through fluorescent microscopy and Scanning Electron Microscopy (SEM). The cells, tissues, and vascular bundles of *C. nocturnum* and rose tissue culture plants were studied in both control and treated plants. AgNPs added at a concentration of 5 ppm to the tissue culture medium of both test plants were safe and effective in controlling the contamination and the bacterial and fungal attack in the tissue culture medium was reduced; it gave the highest percentage of survived plants in both the rose and the cestrum plant. No significant difference was observed in the chlorophyll content of the plants maintained on treatment medium having AgNPs concentration of 5 ppm and the control plants maintained on medium without nanoparticles. The molecular assessment of the stress impact of AgNPs treatment was analyzed through expression of the SAND and PP2A housekeeping genes in treated plants and control plants by comparing their mRNA profile on real-time PCR, and the results showed equal expression of SAND and PP2A in both the control and treated plants. This study proves that low amounts of bio-synthesized silver nanoparticles when supplied in tissue culture media can act as an anti-contaminant in rose and cestrum in vitro cultures without affecting its morphology, physiology, and genetics.

## Introduction

*Cestrum nocturnum L* is part of *Solanaceae* family which is a shrubby plant that is a garden shrub. It has on average 200 different species. Its flowers emit a pleasant smell during the night period so named ‘night queen’. They were native to tropical America and certain regions of the West Indies and usually grow in forests and damp soil but can also be grown in yards and gardens [1]. The leaflets of *C. nocturnum* are extremely significant in conventional healthcare and were used to treat inflammation and wounds as leaves possess analgesic, antibacterial, and antioxidant properties [2]. Additionally, it was documented that the plant has been utilized for local anesthesia, to inhibit the central nervous system, and arrhythmogenic beats of the heart. Higher calcium levels in the blood are triggered by the glycoside that is found in mature leaves of *C. nocturnum*, and is also linked with vitamin D toxication [3]. Its extracts are effective against cancer, additionally, anti-pathogenic and antibacterial activity is seen throughout the entire plant. A wide range of components made from *C. nocturnum* aerial segments were tested to determine their cytotoxic, antibacterial, and antifungal activities [4–7].

Rose (*Rosa indica*) is a woody perennial genus containing up to 100 species, among these, most of the species are found in the regions of Northern Hemisphere having temperate or sub-tropical climates. Because of its beauty and association with special occasions, it is grown worldwide in large quantities. There are approximately 200 million roses planted each year. Vegetative propagation has not been very fruitful for the cultivation of the rose plant [8]. Plant biotechnology offers other methods for rose multiplication. These methods proved helpful for greater yield (a single rose plant might produce as many as 400,000 plants a year), plant preservation, and breeding problems [9]. These cultivation techniques demand high revenue, and the cost can be in vain easily due to the vulnerability of microbial attacks on tissue-cultured plants. The use of disinfectants and fungicides poorly resolves this issue [10].

Nanotechnology is an emerging discipline for creating materials at the nanoscale level. Bio-nanotechnology is a merger of biotechnology and nanotechnology. This technology aims at the production of nature-friendly biosynthetic nanoparticles. There are multiple applications for silver nanoparticles in scientific disciplines. Complementing the nanoparticles in tissue-cultured plants has shown exceptional response [11,12]. NPs when added to the plant tissue culture media, enhance the production of biologically active proteins, trigger seed germination, improve growth, and even impart genetic changes. Nanoparticles are being produced by a variety of medicinal plants and microbes. Rai and colleagues [13] outlined the three primary ways of manufacturing nanoparticles: chemical, physical, and biological. Silver has been utilized as a sterilizing substance since ages ago. Silver nanoparticles are less expensive than those containing gold. In literature, prokaryotic cells are highly susceptible to silver nanoparticles, but eukaryotic cells are not affected by them [14,15].

The major issue that must be fixed is the constant contamination in tissue-cultured plants. Fungi and bacteria are the main causative agents of contamination in tissue culturing of woody plants [16]. Continuous use of chemotherapeutic and antibacterial agents may negatively impact the development of plant culture; thus, they are phytotoxic and detoxicate the development of tissue-cultured plants. Abdi and colleagues [17] first described the protocol in which silver nanoparticles were used in plant growth medium to be used as anti-contaminant agents. In woody trees, micropropagation is mainly affected by internal wounds, and exposure to AgNPs proved to be effective. It is interesting to note that centuries ago, people were also mindful of silver’s antibacterial potential. Studies have proven that the growth of microbial cells is affected by AgNPs and they can also be made a part of disinfectant to be sprayed on explants. AgNPs play an efficient role in the decontamination of the plant culture without having adverse effects on plant cellular growth [18]. Factors like shape, size, dimension, and chemical properties of NPs affect the response. AgNPs are being rapidly used for the improved mineral uptake of plants, enhanced seed germination, explant generation, increased plant biomass, and altered biochemical pathways [19].

Indepth studies have been conducted on subject plants to explore the possible negative impact of AgNPs when applied in plant tissue culture medium as an anticontaminant agent to avoid wastage of elite plants’ in vitro cultures because of biological contaminants. To reach out the stress effect of AgNPs treatments on plant morphology (by comparing growth parameters, physical changes, cytotoxicity, and chlorophyll content) and genetic expression pattern (of housekeeping genes like protein phosphatase 2A, the PP2A; and SAND-family protein, the SAND), comparative analyses have been made between the control and treated samples.

## Materials and methods

The cestrum and rose plants were collected from Yasir Nursery, Lahore. These plants were grown in the trial field area of Centre for Applied Molecular Biology (CAMB), University of the Punjab, Lahore, Pakistan. All of the experiments were performed at the Plant Molecular Biology lab of CAMB except scanning electron microscopy which was performed at a commercial facility of Lahore College for Women University (LCWU), Lahore, Pakistan.

1-Establishment of in vitro cultures Various sections of plants were collected as explant samples to be cultured in a plant tissue culture lab. Fresh samples of cestrum and rose were rinsed under surplus tap water and were surface sterilized following the method described by Daud and colleagues [20] to reduce microbial contamination. Later, they were washed with distilled water thrice and put on blotting paper for sterile air drying. The nodal sections were inoculated in micropropagation medium which was prepared using 4.43g per liter MS (Sigma Aldrich M5519-10L), 30g per liter Sucrose, 2 mg/L BAP; pH adjusted between 5.5–5.7 and 2.5g per liter phytagel was added for gelling purpose. The medium was sterilized by heating at 121^o^C and 105KPas pressure for 20 min in an autoclave. The inoculated cultures were incubated at 20 ± 2°C for 16 light hours and 8 dark hours.

2-Treatment of AgNPs in micropropagation medium Silver nanoparticles (AgNPs) were biogenically synthesized in bulk from callus of *Carica papaya*. We have reported these findings in detail earlier [21]. Healthy cultures (of approximate age and size) of both of the plants, the cestrum and the rose, were maintained on the same medium (MS media supplemented with 2 mg/L of BAP) having different concentrations of silver nanoparticles like 1) 0.002g/L or 2 ppm and 2) 0.003g/L or 3 ppm and 3) 0.005g/L or 5 ppm of silver nanoparticles for two months. The treated cultures were provided the same conditions as of control samples. After exposure, they were returned to the same medium without silver nanoparticles.

### 2.1. Estimation of anti-contaminant potential of AgNPs treatments

Healthy and vigorous tissue cultured plantlets of cestrum and the rose of approximate size and age were shifted to the experimental media having various treatments as described in the previous section. All of the plants were maintained (before shifting to treatment media) on MS medium supplemented with 2 mg/L of BAP. The shifting of plants was carried out under completely aseptic and controlled conditions. Survival percentage was calculated, after 90 days of exposure/incubation, by counting the number of plants that survived contamination and/or discoloration/death in each particular treatment and control group of plants. However, the tissue culture medium was replaced with the same fresh medium every two weeks.

### 2.2. Evaluation of impact of AgNPs stress on physical growth

**Fig 1:**
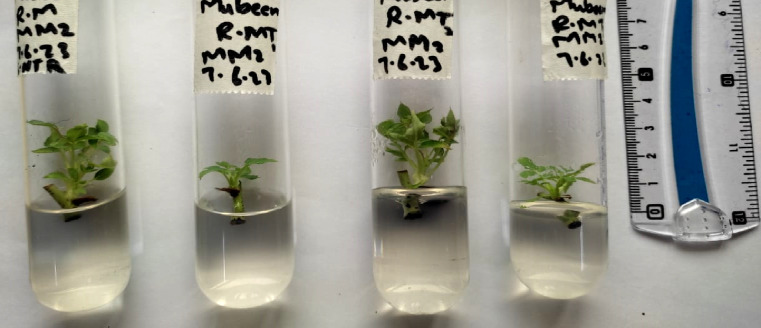
Shoot length measurement in rose plants for assessment of physical growth parameter.

**Fig 2:**
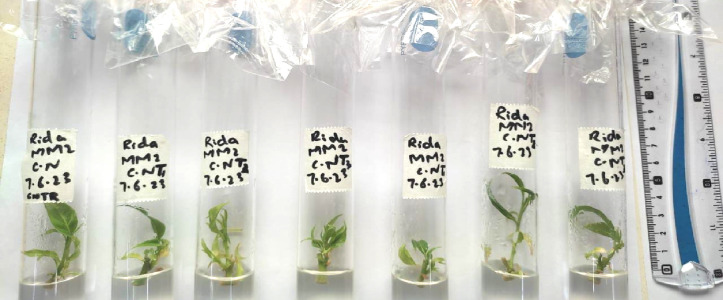
Shoot length measurement in cestrum plants for assessment of physical growth parameter.

Plant growth progress can be assessed in terms of shoot length, root length, fresh weight of plant number of leaves etc. abnormalities in physical appearance and growth can give a basic signal of cytotoxicity inside a plant. Effects of AgNPs were noted in the following terms: shoot length, number of leaves, and physical appearance of micro rose and cestrum plants grown in vitro. The scale was used to measure the shoot length in centimeters of all treated and control tissue cultured plants after 35 days (rose and cestrum plants Fig 1 and Fig 2, respectively). Number of leaves of plants grown in replicates was recorded in data after 35 days. The data values were calculated using standard error and deviation applied in Microsoft Excel.

### 2.3. Evaluation of impact of AgNPs stress on plant cells

Or analysis of stress impact on a single cell, owing to AgNPs treatment, the cross-section and peel-off sections of shoots from treated plants and control plants were analyzed and captured under a light microscope [Nikon, Model No. ECL IPSE Ts2-FL (245,795)]. To study the morphology and anatomy at cellular and tissue level, the cut sections and thin layers from shoots of rose and *cestrum* (grown in media with 5 ppm concentrations of silver nanoparticles), were observed and captured under a scanning electron microscope of a commercial facility at Lahore College for Women University (LCWU). Control plants were also observed and captured for comparison.

### 2.4. Evaluation of impact of AgNPs stress on chlorophyll content

The study used a method to estimate chlorophyll content in control tissue cultured plants and those treated with silver nanoparticles for both the cestrum and rose. The chlorophyll content was checked following the method of Vaishnav and colleagues [22] with some modification (the plant sample was ground, soaked in 80% acetone, and incubated in a dark room for 24 hours). The absorbance was recorded using a UV-Vis Spectrophotometer. Chlorophyll a and b were calculated through the following formulae:


$$\text{Chl}\;\text{a}=\left(12.2^{*}\text{OD}\;{\text{A}}_{\text{663}}\right)-(2.8^{*}\text{OD}\;{\text{A}}_{\text{645}})$$

$$\text{Chl}\;\text{b}=\left(20.1^{*}\text{OD}\;{\text{A}}_{\text{663}}\right)-(5.0^{*}\text{OD}\;{\text{A}}_{\text{645}})$$

Total chlorophyll content=chl a+chl b

### 2.5. Evaluation of the impact of AgNPs stress on PP2A and SAND genes’ expression

For the molecular analysis, plants that were exposed to Treatment-3 (0.005g/L or 5 ppm) were analyzed and compared with control samples. Expression of housekeeping genes PP2A and SAND was analyzed through mRNA profiling. For that purpose ribonucleic acid (RNA) was extracted from fresh samples (young leaf primordia) of rose and cestrum plants following the protocol described by Jahangir and colleagues [23]. mRNA purification and cDNA synthesis was also done opting the method reported by Jahangir and colleagues [23] in the same article. For amplification of PP2A (accession no. JN399224) and SAND (accession no. JN399228) genes, reported primer sequences were used (PP2A forward primer: TGTCACTGCATCAAAGGACAG, PP2A reverse primer: GACGAATTGTCTTCTCCACCCA; and SAND forward primer: GTGTTGAGGAGTTGCCTCTTG and SAND reverse primer: AACCTGTCGGGAGAATCTGTT). Annealing temperatures for the primers of both housekeeping genes i.e., SAND and PP2A were optimized in gradient thermalcycler at a temperature range of 50^o^C-60^o^C. 20µl reaction mixture contained 10µl of 2x Dream Taq Master Mix, 1 picomol each of forward and reverse primers (1.0 µl each), 3.0 µl of cDNA and Nuclease free water (3 µl). The reaction profile was set at pre-denaturation at 95^o^ C (3 min), denaturation at 95^o^ C (45s), annealing from 50-60^o^ C (45s), and extension at 72^o^ C (1 min); with 35 repeats of the amplification cycle and final extension of 72^o^ C for 7 min. PCR products were run on 1.5% agarose gel at a rate of 5volts per centimeter for 40 min.

After optimization of cycling conditions, real-time PCR was run to analyze the mRNA profiling of SAND and PP2A housekeeping genes. 25µl of reaction mixture was prepared from 2X SYBER Green Master mix (12.5 µl), 1 picomol each of forward and reverse primers, and 3 µl of cDNA of rose and cestrum in separate reaction sets. For amplification, optimized cycling conditions were practiced; which had initial denaturation at 95^o^ C (3 min), denaturation at 95^o^ C (30s), annealing from 55^o^ C (30s), extension at 72^o^ C (30s) and final extension at 72^o^ C (7 min). Extracted and purified nucleic acid, and amplified PCR products were checked by resolving on 1% high melt agarose and ethidium bromide using 6X DNA loading dye (1X final fraction in sample) and suitable DNA marker (Lambda Hindi III for nucleic acid and bp DNA ladder for PCR product) was run at a constant voltage of 6volts per centimeter of gel tank length.

## Results

### 1. Establishment of in vitro cultures of cestrum and rose

**Fig 3:**
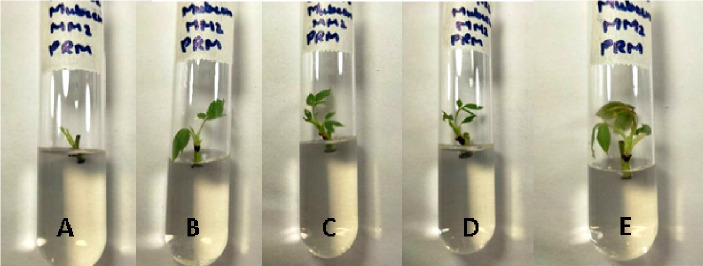
In vitro cultures of rose plants established from nodal explant in MS media supplemented with BAP 2 mg/l captured during micro propagation after 5, 10, 15, 20 and 25 days of inoculation (A to E) respectively.

**Fig 4:**
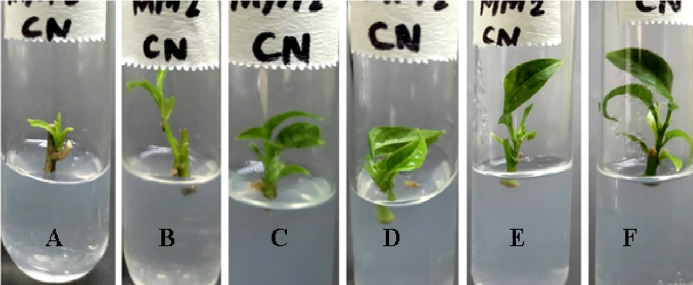
In vitro cultures of Cestrum plants established from nodal explant in MS media supplemented with BAP 2 mg/l captured during micropropagation after 5, 10, 15, 20, and 25 days of inoculation (A to E) respectively.

The microplants produced from nodal sections in micropropagation media supplemented 1 mg/l BAP and 2 mg/l BAP. However better results were observed in a medium containing 2 mg/l BAP as the first leaf primordium emerged only after 5 days of incubation in both plants, the cestrum and rose. Supplementation of BAP hormone in MS media at the concentration of 2 mg/l supported enhanced shoot propagation, boosted growth, and plants uttered multiple shoots and sturdy leaf growth in rose and cestrum (Figs 3 and 4, respectively).

### 2. Determination of anti-contaminant potential of AgNPs treatment on in vitro cultures of Cestrum and Rose

After the shifting of healthy cultures to various treatments of silver nanoparticles, various aspects of treatment effects were studied after a specified period (as described in the methodology and following sections of results). Some plants were maintained on various treatments for a longer time (90 days). The cultures that were maintained in various treatments remained free of contamination even after several attempts of shifting to fresh medium; however, their lush green color turned light and did not increase in size. The plants from Treatment-3 turned about yellow and we considered those as dead. A total number of control cultures (maintained on medium without AgNPs) decreased after every shift due to microbial contamination, however, those remained fresh green, and healthy. The loss of control group cultures due to contamination was higher than the cultures in all treatments; even higher than the cultures in Treatment 3 (Table 1).

**Table 1:** Determination of anti-contaminant potential of AgNPs treatment on in vitro cultures of Cestrum and Rose: a percentage calculated after 90 days of incubation in control medium MS media supplemented with BAP 2 mg/l and same medium having AgNPs in concentration of 1, 3, and 5 ppm as Treatment I, 2 and 3 respectively.

	Total no. of plants studied		Total no. of plants survived		Survival percentage	
	Cestrum	Rose	Cestrum	Rose	Cestrum	Rose
**Exposure to Treatment-1**	70	75	57	59	81.42%	78.66%
**Exposure to Treatment-2**	75	75	70	69	93.33%	92%
**Exposure to Treatment-3**	75	75	49	46	65.33%	61.33%
**Control (nontreated) plant group**	80	85	43	46	55%	54.11%

### 3. Determination of stress effect of AgNPs treatment on Cestrum and rose plants

#### 3.1. Impact of AgNPs on morphological and growth parameters.

**Fig 5:**
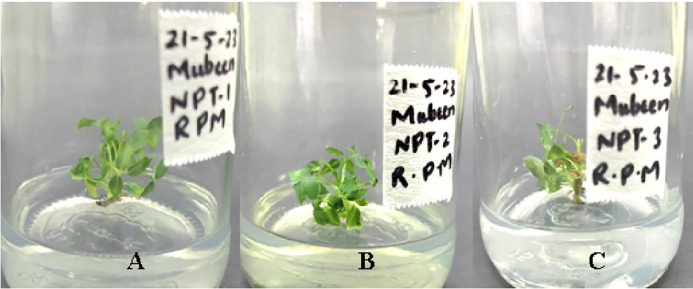
Rose micro plants exposed in AgNPs treatments: (A) Treatment-1, 2 ppm of AgNPs; (B) Treatment-2, 3 ppm of AgNPs; and (C) Treatment-3, 5 ppm of AgNPs; was in MS media supplemented with BAP 2 mg/l.

**Fig 6:**
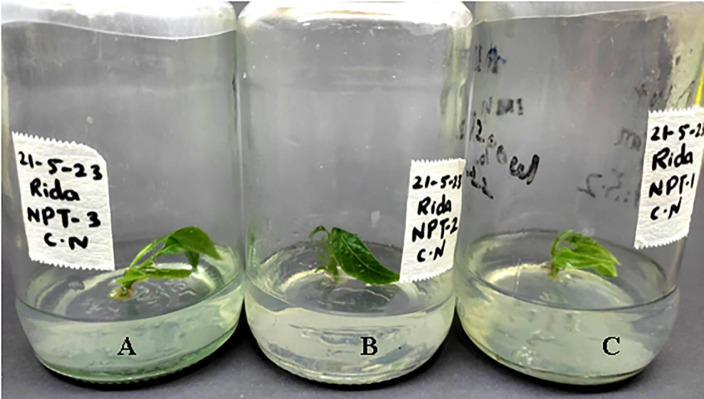
Cestrum micro plants exposed in AgNPs treatments: (A) Treatment-1, 2 ppm of AgNPs; (B) Treatment-2, 3 ppm of AgNPs; and (C) Treatment-3, 5 ppm of AgNPs; was in MS media supplemented with BAP 2 mg/l.

**Fig 7:**
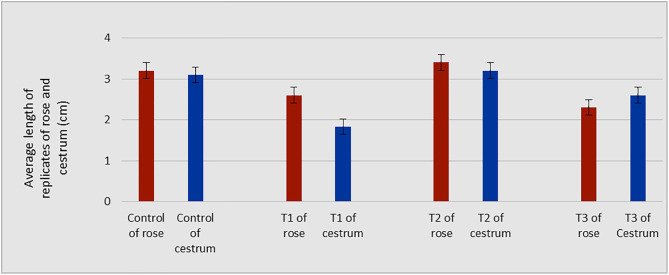
Comparison of shoot length of control and treated plant groups of rose and cestrum: The average of shoot lengths of all replica plants of the control group are compared with the average shoot lengths of all replica plants in three treatments i-e. T1, 2 ppm of AgNPs; T2, 3 ppm of AgNPs; and T3, 5 ppm of AgNPs added in MS media having BAP 2 mg/l.

**Fig 8:**
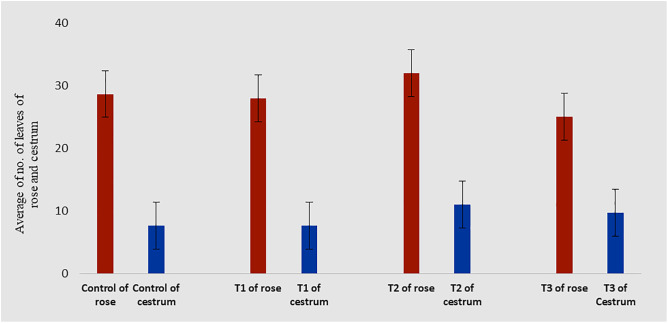
Comparison of leaf count of control and treated plant groups of rose and cestrum: The average of no of leaves of all replica plants of the control group is compared with the average of no of leaves of all replica plants in three treatments i-e. T1, 2 ppm of AgNPs; T2, 3 ppm of AgNPs; and T3, 5 ppm of AgNPs added in MS media having BAP 2 mg/l.

The morphological appearance of cultures incubated in treatments of silver nanoparticles was normal and indiscriminate (in all three concentrations 0.002g/L, 0.003g/L and 0.005g/L) from the control cestrum and control rose plants even after one month of exposure. The treated plants showed the same physical health as the control plants of rose and cestrum as shown in Figs 5 and 6, respectively. They were free of contamination and grew lush green and healthy. All three treatments exhibited anti-contaminant behavior; the plant cultures remained free of any bacterial infection or fungal hyphae. Silver nanoparticles show targeted toxicity as they controlled the microbial cells and inhibited their growth but did not damage the plant cells. The average of shoot (plant) lengths that were measured in various treatments was approximate to each other except in Treatment-2, which was slightly higher than other treatments and the average of control plants (Fig 7). The leaves of treated plants were also compared with the leaves of control plants. Both plant groups were physically comparable in all aspects like color, shape, and size of leaf. Leaf numbers were monitored and recorded for each treatment for both the plants, the rose and cestrum. The highest leaf count was observed in Treatment Media 2 which contained 3 ppm of AgNPs (Fig 8). Number of leaf count was comparatively lower in treatment-3 which contained 5 ppm silver nanoparticles (Fig 8).

#### 3.2. Impact of AgNPs on chlorophyll content.

**Fig 9:**
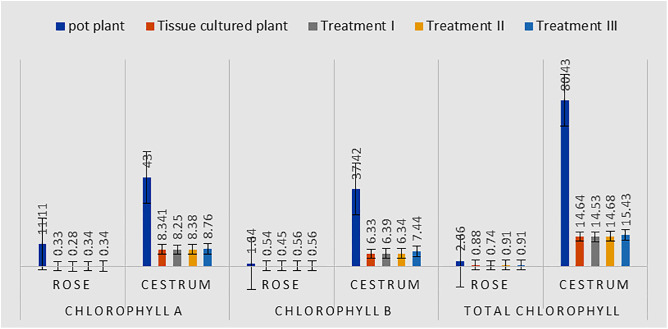
Comparison of chlorophyll content in control and treated plant groups of rose and cestrum: chlorophyll a, chlorophyll b and total chlorophyll content of all replica plants of the control group and the replica plants in three treatments i-e. Treatment-I, II and III (2, 3 and 5 ppm of AgNPs, respectively) are measured and compared with the chlorophyll content of control plants growing in the natural environment.

To identify any cytotoxic effect of silver nanoparticles on the plant’s biological metabolism, we have measured the chlorophyll a, chlorophyll b, and total chlorophyll content. The average values of all replicate plants for a particular plant group have been compared. It was observed that the average values of chlorophyll a content (obtained from the group of) rose cultures exposed to Treatment-1 (0.28) were approximately equal to the average of replicates that were grown in Treatment-2 (0.34) and Treatment-3 (0.34). These values were almost equal to the average value obtained from the group of control rose in vitro cultures (0.33). Similarly, the average values of chlorophyll a content from replicate plants of cestrum for Treatment-1, Treatment-2, Treatment-3, and control lab cultures were recorded as 8.25, 8.38, 8.76, and 8.34 respectively. A similar trend was also observed in the chlorophyll b content and total calculated chlorophyll content in both plants. The value of chlorophyll b and total chlorophyll content of cestrum was higher in those plants that had 5 ppm concentration of AgNPs as compared to other treatments, the 2 and 3 ppm. chlorophyll b content in Rose for Treatment-1, Treatment-2, Treatment-3, and control cultures was recorded as 0.45, 0.56, 0.56, and 0.54 respectively, and chlorophyll b content in cestrum for Treatment-1, Treatment-2, Treatment-3 and control cultures was recorded as 6.39, 6.34, 7.44, and 6.33 respectively. Total chlorophyll content for Treatment-1, Treatment-2, Treatment-3, and control cultures was calculated as 0.74, 0.91, 0.91, 0.88, and 14.53, 14.68, 15.43, 14.64 in rose and cestrum respectively. However, there was a huge difference in the values of chlorophyll a, chlorophyll b, and total chlorophyll content that was measured in samples taken from rose and cestrum plants from an open environment (these served as sources of explants for the production of micro-propagated plants). These observations have been depicted graphically in Fig 9.

#### 3.3. Impact of AgNPs stress on plant cells and tissues.

**Fig 10:**
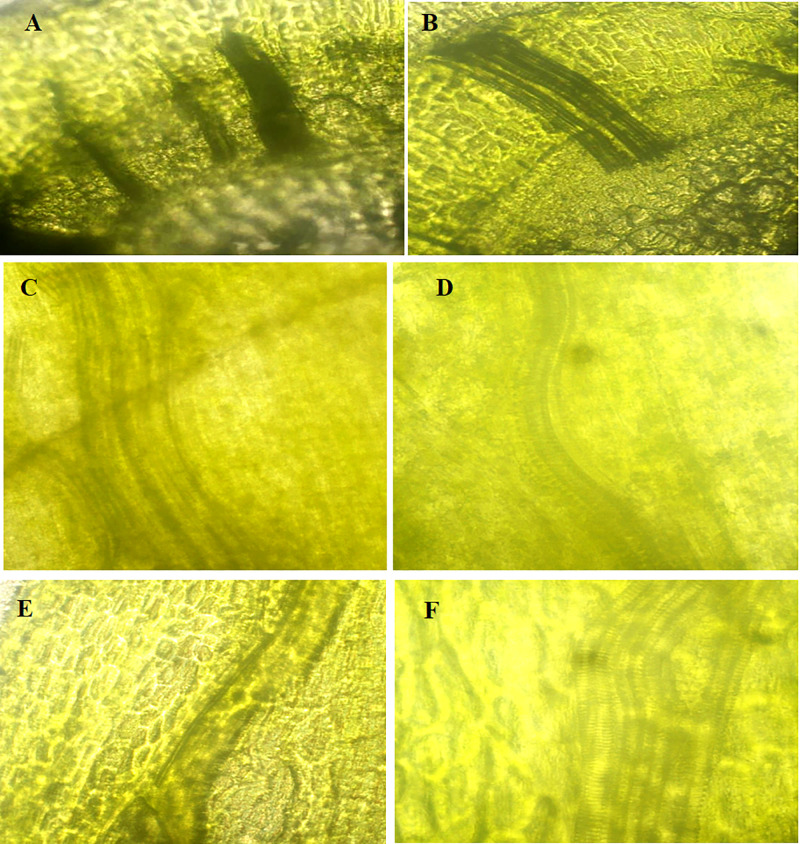
Analysis and comparison of morphology of cells and tissues of cestrum plants from control group and treatment-3 (5 ppm AgNPs) at fluorescent microscope [Nikon, Model No. ECL IPSE Ts2-FL (245,795)].: (A) transverse section of control plant at 40X resolution; (B) transverse section of treated plant at 40X resolution; (C and E) longitudinal section of control plant at 20X and 40X resolution respectively (D and F) longitudinal section of treated plant at 20X and 40X resolution respectively.

The possible impairment caused due to exposure to highest used concentration of AgNPs (Treatment-3, 5 ppm), the transverse and longitudinal thin slices were observed under various magnifications of fluorescent microscope and revealed no significant visible morphological discrimination in the cells and vascular bundles of control and experimental tissue cultured plants of cestrum and rose (Fig 10,11 respectively). The morphology and shape of cells and tissues of control and AgNPs treated microplants have also been observed under SEM and no discrete difference in cellular morphology in analyzed plant groups. The tissues, cell surfaces, and stomata of the peel piece from the control cestrum plant (Fig 12A, C, E, G) are very clearly identical to the issues and surfaces of treated cestrum plant (Fig 12B, D, F, and H). Furthermore, no abnormality or difference is visible in the micrographs of the treated sample. Vascular tissues and cells were very clear and intact in fresh slides of cestrum control and treated slices. However, the scanning electron micrographs of about 30 hours old slides showed that the cestrum tissue was shrunken perhaps due to transpiration and drying of the milky liquid fluid of cestrum cells. However, even then there was no visible difference in the samples from control and treated plants. Figs 13 and 14 depict scanning electron micrographs of experimental and control plants of Rose cross verse section and peel respectively.

**Fig 11:**
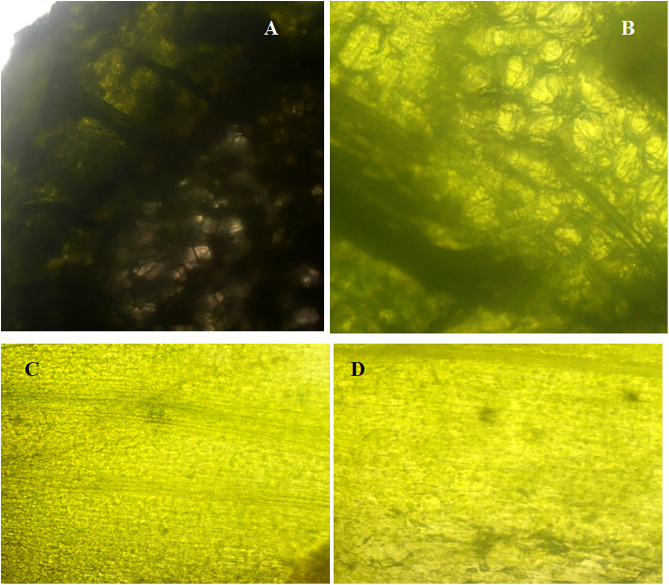
Analysis and comparison of morphology of cells and tissues of rose plants from control group and treatment-3 (5 ppm AgNPs) at fluorescent microscope [Nikon, Model No. ECL IPSE Ts2-FL (245,795)].: (A) transverse section of control plant at 40X resolution; (B) transverse section of treated plant at 40X resolution; (C) longitudinal section of control plant at 20X; (D) longitudinal section of treated plant at 20X resolution.

**Fig 12:**
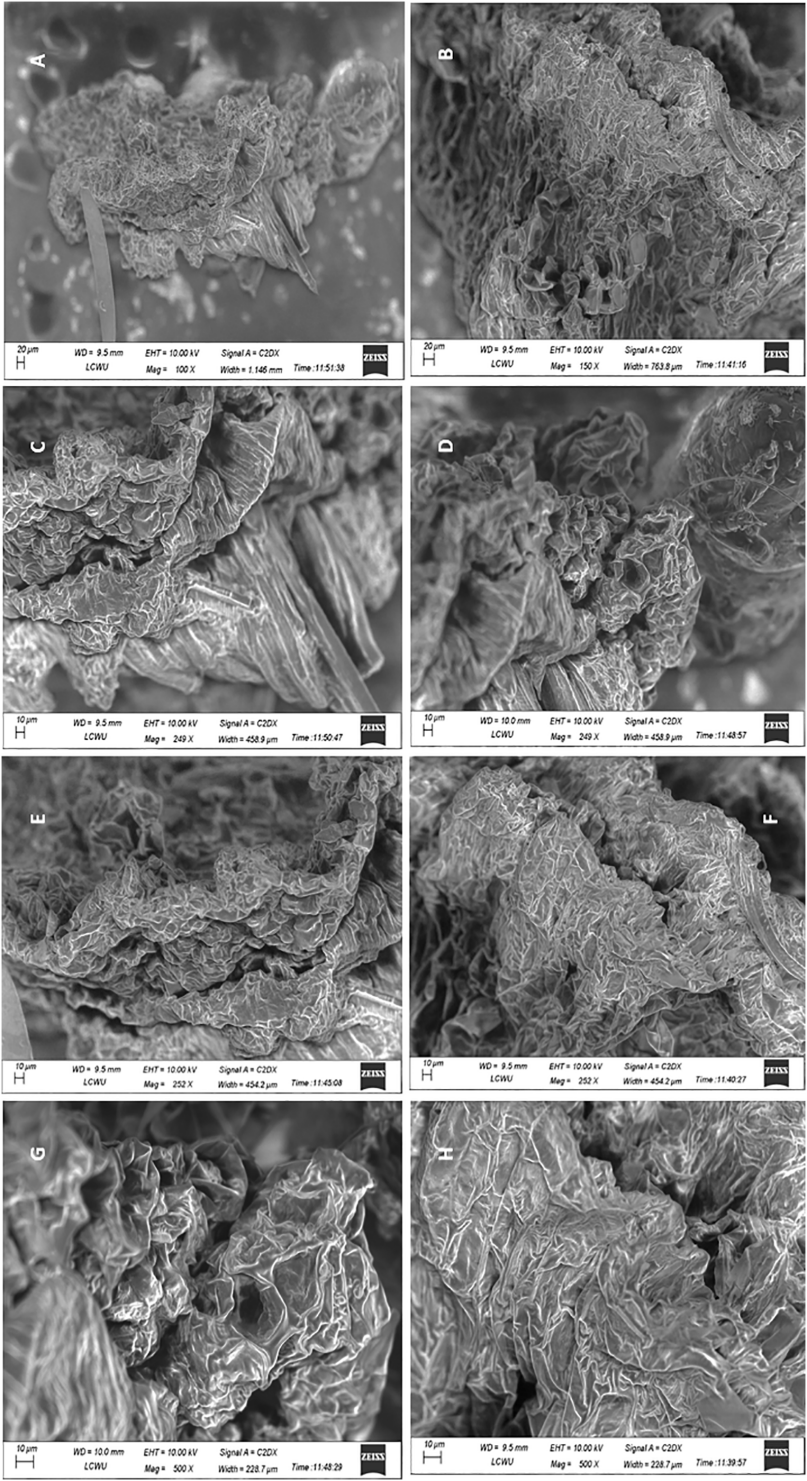
Analysis and comparison of the morphology of cells and tissues of cestrum plants from the control group and treatment-3 (5 ppm AgNPs) a scanning electron microscope (ZEISS).: (A, C, E, G) micrographs of peel of control plant at 100X, 249X, 252X, 500X, 300X, 500X, and 750X magnifications respectively; (B, D, F, H) micrographs of peel of treated plant at 150X, 249X, 252X, 500X, 500X, 500X, and 1000X magnifications respectively.

**Fig 13:**
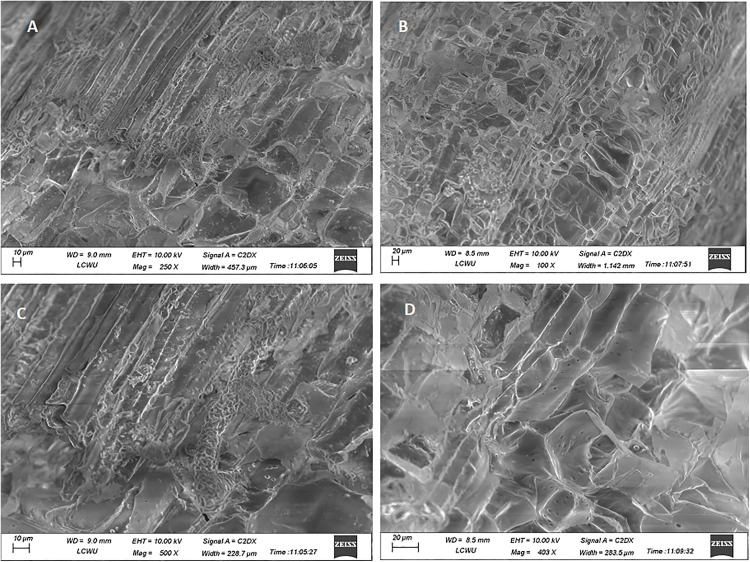
Analysis and comparison of the morphology of cells and tissues of rose plants from the control group and treatment-3 (5 ppm AgNPs) a scanning electron microscope (ZEISS).: (A, and C) micrographs of the cross verse section of the control plant at 250X, and 500X magnifications respectively; (B, and D) micrographs of the ross verse section of a treated plant at 100X and 403X magnifications respectively.

**Fig 14:**
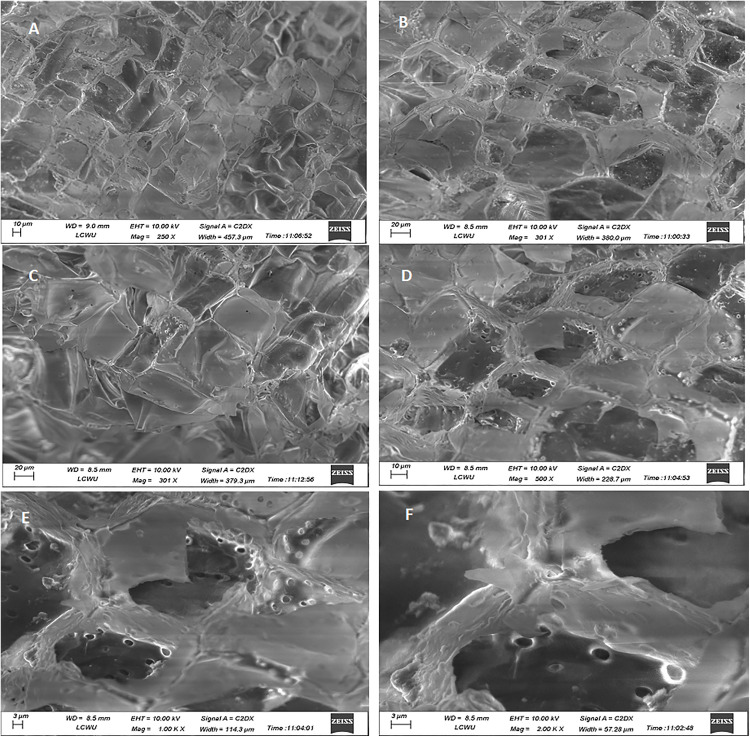
Analysis and comparison of the morphology of cells and tissues of rose plants from the control group and treatment-3 (5 ppm AgNPs) a scanning electron microscope (ZEISS).: (A, and C) micrographs of a longitudinal slice of a control plant at 250X, and 301X magnifications respectively; (B, D, E, and F) micrographs of a longitudinal slice of a treated plant at 301X, 500X, 1000X and 2000X magnifications respectively.

### 3.4. Impact of AgNPs stress on housekeeping gene expression through mRNA profiling

**Fig 15:**
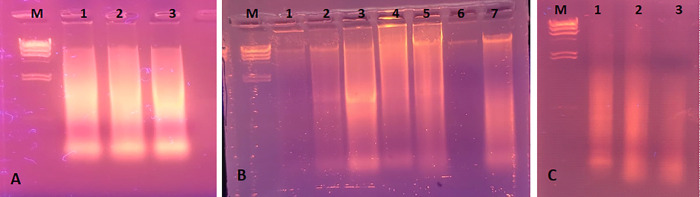
RNA extraction and purification: (A) rose RNA from control (1) and treated plants (2 and 3); (B) cestrum RNA from control (1 and 2) and treated plants (3 to 7); (C) purified RNA of rose control (1) and cestrum control (2,3); M is Lambda Hindi III DNA marker.

**Fig 16:**
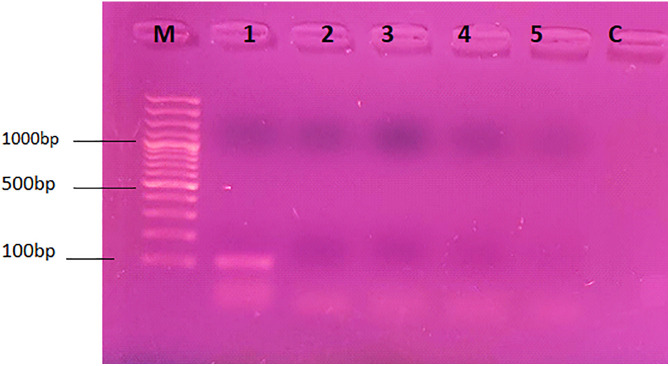
PCR conditions (annealing temperature) optimized for SAND gene primers: M is 100 bp DNA ladder, 1 PCR with annealing at 55^o^C, 2 at 56^o^C, 3 at 57^o^C, 4 at 58^o^C and 5 at 59^o^C, and C is PCR negative control.

**Fig 17:**
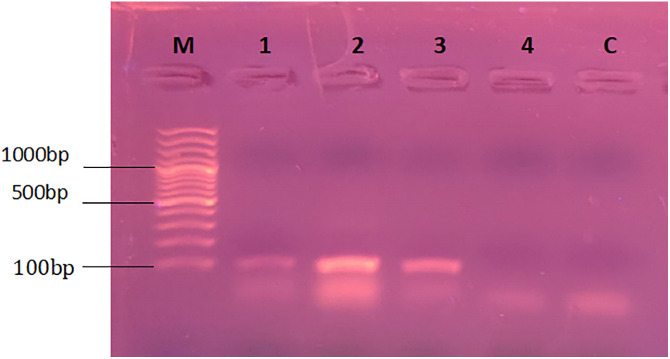
PCR conditions (annealing temperature) optimized for PP2A gene primers: M is 100 bp DNA ladder, 1 PCR with annealing at 54^o^C, 2 at 55^o^C, 3 at 56^o^C, 4 at 57^o^C, and C is PCR negative control.

**Fig 18:**
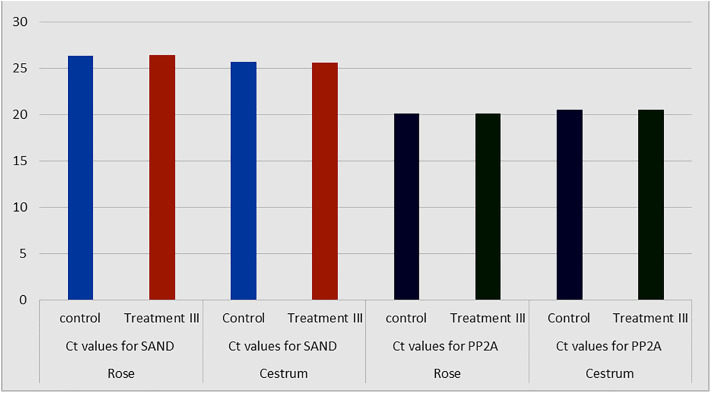
Comparison of gene expression between treated and control plant groups of rose and cestrum: Ct values (Average of 10 replicates) obtained for rose and cestrum of treated (5 ppm) and control plants for SAND and PP2A gene.

Whole genome nucleic acid was successfully extracted (Rose and Cestrum in Fig 15 A and B respectively) and purified (Figs 15 C), from tissue-cultured plants of the control group and 5 ppm treatment. Before the experimentation of mRNA profiling, the annealing temperature of the SAND and PP2A gene primers was optimized with the cDNA of the control plant at a conventional thermal cycler and revealed as 55^o^C for PP2A and SAND gene primers (Figs 16 and 17 respectively). The comparison of gene expression was made based on Ct values of SAND and PP2A genes’ amplification and detection in real time reactions of control replicates and treated (5 ppm) replicates. The measurement of intensity of fluorescence emitted by the probe at each cycle was measured by qPCR machine. The Ct measure is a determined PCR cycle that represents the basic result of qPCR experiment. The lower Ct values, the greater the expression of housekeeping genes observed. It was observed from the Ct values of SAND and PP2A genes that the expression of both genes is slightly different from each other, however, the Ct values of either gene were about identical in the replicates of the control plant group and experimental plants in both tested plants. Fig 18 graphically explains the comparison of the average of Ct values (Average of 10 replicates) obtained for SAND and PP2A gene in Rose and Cestrum control plants and plants from (5 ppm) Treatment.

## Discussion

### 1. Establishment of in vitro culture *of* rose and cestrum

Among various hormonal combinations and concentrations tested like BA (0.5 mg/L), BA (1 mg/L), IBA (1 mg/L), BA and NAA (1.5 mg/L) respectively, the MS medium having BA (2 mg/L) concentration proved most effective and efficient for propagation of microplants from the nodal meristematic region and the shoot apical meristem of rose and cestrum. These findings are very close to the work reported by Sarkar and colleagues [24] and Shabbir and colleagues [25], who micro-propagated using its shoot-tip as an explant in media supplemented with BA (1.5 mg/L). However, these outcomes contradict the Abubacker and colleagues [26] findings, who used BA (2 mg/L) in MS media with the midrib. And, they didn’t find satisfactory growth results in the above said hormonal concentration from midrib. Chhalgri and colleagues [27] also used BAP hormone for micropropagation in MS media (using a mixture of 3 mg/l IAA and 3 mg/l BAP) for the propagation of shoots from the nodes of the rose plant. In a different experiment, MS media containing 0.1 mg/l GA3, 0.5 mg/l kinetin, and 2 mg/l BAP was utilized and obtained 100% shoot elongation from nodal zones of the rose plant [8,28,29].

### 2. Determination of anti-contaminant potential of AgNPs treatment on in vitro cultures of Cestrum and Rose

The anti-contaminant capacity of silver nanoparticles on the growth of rose and cestrum explants was assessed and found 0.003g/L or 3 ppm was the most effective and safe concentration of silver nanoparticles in cestrum. At this amount, a great deal of the contamination has been controlled along with improving physical condition, length of shoot, leaf number as well biomass, and stomatal activity. Several AgNP ratios have been employed, including 2, 3, and 5 ppm. The findings reported by Mahajan and colleagues [30], who used 0.03g/L concentration of AgNPs, strengthen our results. Mahajan and colleagues [30] claimed that the lower the concentration of AgNPs, the better the results. However, our findings are contradictory to Gouran and colleagues [31] who utilized 1g/L concentration of AgNPs in tissue cultured medium to reduce internal contamination. Tung and colleagues [32] grew *Chrysanthemum* apical meristems on a culture medium supplied with 4 ppm AgNPs and proved that the meristems were preserved. They claimed treatment with a higher concentration of AgNPs was more effective. Their interpretation also follows our conclusion that a higher concentration of AgNPs was more effective and safer (Table 1) but this is true only for a limited time of treatment like a few months. Longer exposure of plants to higher concentrations of AgNPs causes growth retardation and even plant death (Table-1). Our inference that prolonged exposure to higher concentrations of AgNPs harms plants is strengthened by the findings of Sarmast and Salehi [33] who experimented on the tobacco plant to assess anti-microbial potency of silver nanoparticles. They used high doses of NPs and found a prominent antimicrobial effect in treated plants, but the sub-lethal dose disrupted metabolic pathways in the treated plants [33].

### 3. Determination of stress effect of AgNPs treatment on Cestrum and rose plants

#### 3.1. Effect of AgNPs treatment on plant growth and morphology.

It is clear from the presented experiments that MS media enriched with 3 ppm concentration of AgNPs is not only safe rather growth-promoting as well and caused the increased size (up to 5.2 cm in *C. nocturnum* plant) and the number of shoots (Figs 7–8). This addition resulted in the increase of shoot length and number of leaves up to 6 cm and 12. These findings are close to the Elsayh and colleagues [34] who reported that shoot number and leaf number was increased to 10.74 and 15 at 0.003g/l concentration of AgNPs. In our findings, the length of the shoot (and leaf) was also increased up to 6.60 cm (Figs 7–8). However, these findings were contradicted by the findings of Timoteo and colleagues [35] who observed that shoot numbers remained the same before and after the treatment of AgNPs. They further explained that they found no significant difference in the number of shoots and leaves before and after the treatment. They concluded that AgNPs do not influence the growth of tissue-cultured plants. Their interpretation does not support our findings of the growth-promoting effect of AgNPs; perhaps it is due to differences in the signal response by individual plant species. Boosted shoot length (being reported here in Fig 7) in rose micro-cultures supplied with silver nanoparticles in their growth medium, is similar to the findings of Aghdaei and colleagues [36], as their study implicated a direct impact on the shoot length when ethylene blocker was added along with the NPs. The fresh shoots still grew well, and the plant survived. Studies reported by Krishnaraj and colleagues [37] and Baskar and colleagues [38], also revealed the growth-stimulating effect of AgNPs on plants. Pallavi and colleagues [39]  targeted three edible plants: *Brassica,* wheat, and cowpea to study the effect of AgNPs, the nanoparticles were sprayed in three concentrations (0, 50, and 75 ppm). Their results showed that wheat plants didn’t get affected at any concentration, while in cowpea root nodes were boosted at 50 ppm increased and shooting was enhanced in *Brassica* at 75 ppm silver nanoparticle concentration. In a recent study reported by Mustafa and colleagues [40], on soybean to find out the molecular and physiological impact of biosynthetic silver nanoparticles in comparison to chemically synthesized NPs. Their findings proved that biologically synthesized nanoparticles were able to boost the length of the root and the hypocotyl.

#### 3.2. Effect of AgNPs treatment on plant chlorophyll content.

In this study, the total chlorophyll content of Cestrum tissue cultured plants that have been treated with silver nanoparticles is increased by using 80% acetone. The value of chlorophyll b and total chlorophyll content of Cestrum was higher in those plants that had with 5 ppm concentration of AgNPs as compared to other treatments (Fig 9). Similar observations have also been reported in the study published by Castro-González and colleagues [41]; their results showed total chlorophyll considerably increased at doses of 25, 50, and 100 mg/L of AgNPs in wheat-established in vitro conditions. Our findings are also very close to the results reported by Verma and colleagues [42] who experimented to estimate the chlorophyll content after AgNPs treatment in plants. He explained that the photosynthetic pigment of AgNPs treated plants was greater in 80% acetone. However, a contradiction is found with the findings reported by Sumanta and colleagues [43]; they used various solvents to estimate the photosynthetic pigment. They further concluded that Di-ethyl ether was good for chlorophyll extraction and estimation.

#### 3.3. Effect of AgNPs treatment on plant cells and tissues.

The Fluorescent micrographs of transverse and longitudinal sections of control and treated rose and cestrum plants have revealed that the vascular bundles and other tissues and cells have no visible difference in control and treated samples that were analyzed (Figs 10–14). The study reported by Cvjetko and colleagues [44] supports our methodology (Figs 10,11) because they have observed the subcellular compartments (vacuoles of root cells) of AgNPs treated plants and control plants at 1, 3, and 5µm at fluorescent microscope. Khatoon and colleagues [45] also used the fluorescent microscope for structural analysis of plant tissue. However, their findings do not support our claim that AgNPs treatment (in tissue culture medium) for protection of plants from microbes does not harm plant cells and tissues (Figs 10–14) as they showed a slight change in control and AgNPs treated tissue cultured plants at 300–360nm.

We have analyzed the control and treated plants (after 35 days of treatment with 5 ppm AgNPs) under various magnification powers of scanning electron microscope ranging from 100X to 2000X for structural comparison (Figs 12–14). Timoteo and colleagues [35] have also used 90 days older tissue cultured plants for SEM analysis at 20X resolution and claimed that both treated and control plants have more prominent stomata on leaves. On the other hand, various shapes of cells i.e. spherical and oval of AgNPs treated plants at 5000X resolution of SEM were reported by Verma and colleagues [42]. Subramaniam and colleagues [46] analyzed AgNPs treated tissue culture plants at the 500X resolution of SEM. In the presented work, surface area, nature, particle range, and pore size were analyzed at 2000X magnification, and found no abnormality (Fig 14). We have found no morphological changes between the control and experimental samples were observed after 35 days of exposure even when the AgNPs were added at 5 ppm concentration.

### 3.4. Effect of AgNP treatment on gene expression

The impact of Treatment on the expression profile of SAND and PP2A housekeeping genes in Rose and Cestrum tissue cultured plants has been observed on Real Time PCR. For the Cestrum plant it was observed from the C_t_ values that the gene expression is prominently alike in the control replicates and the replicates of treatment-3, the 5 ppm concentration of AgNPs. Lower C_t_ values in Real Time PCR represent the earlier detection and higher expression of the genes being analyzed. In our analysis, we found unaffected and comparable expression of SAND and PP2A housekeeping genes in control and treated Cestrum plants (12 replicate plants analyzed) even after 35 days of exposure (Fig 18). While some other researchers like Ramezani and colleagues [47] have linked the higher concentration of AgNPs treatment with enhanced expression of the housekeeping gene in comparison with the control plant. They have further explained the low level of gene expression in the lower concentration of AgNPs treatment, up to 10mM. Another study, reported by Selvakesavan and colleagues [48], has found a linkage and dependence of the expression of housekeeping genes on the duration of AgNPs treatment. They observed higher C*t* values in longer duration of treatment, which means they got lower expression of housekeeping genes with longer exposure to AgNPs.

We have found an insignificant impact of silver nanoparticles’ treatment on the expression of SAND and PP2A housekeeping genes in Rose plant. We have not observed any huge difference in the C*t* values obtained from the control and the treated plants (10 replicates each; Fig 18). Other reported data show a clear contradiction from our outcomes, Kaveh and colleagues [49] found that the experimental plants exposed to 20nm silver nanoparticles for 10 days of exposure caused upregulation of 286 genes and the suppression of 81 genes in *Arabidopsis thaliana*. The pot plants, on the treatment of 10 days of silver ions showed 84 genes were upregulated and 53 genes were suppressed. They have used PVP-coated silver nanoparticles and silver ions to analyze changes in the expression of genes in *Arabidopsis thaliana* through Affymetrix expression microarrays. Salesa and colleagues [50] also reported the impact of AgNPs on the upregulation of *SOD1* and *MMP1* by evaluating 13 different genes. However, some studies reported chances of genetic disruption as observed by Kumari and colleagues [51] where they stated that AgNPs infiltrate the plant’s cellular system and disrupt proper internal metabolism ultimately reducing cellular reproduction. When treated with 100 ppm concentration of nanoparticles the mitotic index reduced from 60.3% to 27.62%. The cellular division ceased causing chromosomal loss. These findings implicate the importance of assessing the universal destination, and the route of AgNPs in the cell metabolic pathways especially in an open environment. All of the above-quoted findings are entirely contradictory to our reported findings there might be two possible reasons behind this contradiction. First, we used very low concentrations of silver nanoparticles as treatment because we were focused on analyzing the anti-contaminant and plant protective impact of AgNPs when added to the tissue culture medium. A second possible reason is the individual response of plant species to the stress signal of AgNPs treatment.

## Conclusions

The presented study is the continuity of our earlier published work [21]. The antimicrobial potential of silver nanoparticles biogenically synthesized from callus of *Carica papaya* is also well established. In the current study, in vitro cultures of Rose and Cestrum have been successfully grown in MS media supplemented with 2 mg/L BAP. We have checked the plant protective potential of the silver nanoparticles when added to the tissue culture medium of Rose and Cestrum plants. We have found that biogenically synthesized AgNPs are safe and effective anti-contaminant sources in the tissue culture medium up to 5 ppm concentration and 35 days of exposure. It is revealed that 3 ppm concentration of AgNPs can successfully defend in vitro cultures of Rose and Cestrum from general lab contamination without posing any stress impact on the morphology and growth of exposed tissue cultured plants. Rather, 3 and 5 ppm concentrations improved the health and growth parameters of plants. Microscopy and scanning electron microscopy revealed that the physical health of internal tissues and cells of AgNPs treated plants remain unaffected and normal as of control tissue cultured plants. No stress impact has been observed on the expression of SAND and PP2A housekeeping genes in AgNPs-treated Rose and Cestrum plants. Based on C*t* values of real-time PCR, identical expression profiles have been obtained in the AgNPs treated and control Rose plants; similarly, in treated and control Cestrum plants. Thus, biologically synthesized AgNPs can safely be used as plant protective and anti-microbial agents in plant tissue culture laboratories to avoid contamination loss of precious experimental germplasm.

### Future perspectives

It is believed that 5 ppm concentration of silver nanoparticles can be used in tissue culture media to prevent contamination in Rose, Cestrum, and other plants in the future. In rose and *C. nocturnum* plants, AgNPs’ potential against tissue culture contamination had not been documented before the presented study. Various doses of *silver* nanoparticles can be used in tissue culture medium to break dormancy and for improved physical health in terms of growth, development, better shoot length, enhanced leaf count, and better physiological aspects.
